# Supplementary material for: Patterns of Intron Gain and Loss in Fungi
Source: PLoS Biol. 2004 Nov 30;2(12):e422. doi: 10.1371/journal.pbio.0020422 (PMC532390; doi:10.1371/journal.pbio.0020422)
Supplement: Table S1 — Also available at http://genes.mit.edu/NielsenEtAl/. (4.3 MB ZIP). [file pbio.0020422.st001.zip › NielsenEtAl/html/1424.html]

AN7293.1.NCU07563.1.MG00127.1.FG06758.1


```
 CLUSTAL W (1.82) Multiple Sequence Alignments - Introns Inserted


Sequence 1: MG00127.1	429 aa
Sequence 2: FG06758.1	421 aa
Sequence 3: NCU07563.1	439 aa
Sequence 4: AN7293.1	430 aa
Alignment Length: 462 aa
Number Identitical Residues: 98 aa
Alignment Score (without introns) 6374


MG00127.1 	MPTATSKHHHGRQYDIVVFGATG~YTGVFTAEQVAATLPTDVNWAIAGRSQEKLQKIATD
NCU07563.1	----MPLKQHGRDYDIVVYGASG1YTGKYTAQHIATHLPTTLKWAVAGRSRSKLEAVVSR
FG06758.1 	----MALKDHGRQYDIVIFGATG1YTGKLTAEYIATHLPTDLKWAVAGRNESKLDALVED
AN7293.1  	-------MESSREFDIVVLGPTG~YTGKYCAEHIVTHLPTNLKWALAGRSTKKIEGVAQE
          	        . .*::***: *.:* ***   *: :.: *** ::**:***. .*:: :.  

MG00127.1 	LKAKYPDRRQP---------------A1IEVCN--LNDQDLTTLVKRTFVLLATVGPYSH
NCU07563.1	LQELNPDRLPP---------------S~IEIISSTTDRTALEALCRRTFILLTTVGPYGS
FG06758.1 	CKKRNSDRLQP---------------A1IEIAN--LNDADLSALAKKTCVLLTTVGPYSL
AN7293.1  	LKKLNPDRLDPGKTMYWCLWSGSADAS~IEIISVQLNKEELRSLAERTKLIINCVGPYHI
          	 :   .**  *..:   .  :.::.:: **: .   :   * :* .:* :::  ****  

MG00127.1 	YGEYAFKACAQNGTHYLDV~TGELAWTSIMIKKYEDTAKATGAI~MIPQIGFESAPADLI
NCU07563.1	LGEPAFAACATTGTHYFDV~TGEVPFVHKMITKYSSLAAQSGAK~MFPQIGIESAPSDLL
FG06758.1 	YGEHAYKACAEEGTHYVDV~TGEAAWVHKMIKKYEATAKKTGAI~LIPQAGIESAPADLI
AN7293.1  	YSTPVVEACANAGTHYVDA2TGETPWVKQTVDKYHGTAKSNGAI0IIHCVGIESAPADML
          	 .  .  ***  ****.*. *** .:.   : **   *  .**  ::   *:****:*::

MG00127.1 	TFALAKAVREELNAPTRD--VRLSVDMTN~P-FPSGGTWLSVFAMLDLFSLKLMAELSKP
NCU07563.1	TWSLTQQLKREFGPATKTGQVTLSIHTLR~S-APSGGTLATVFTILETFSLKQLKEAYKP
FG06758.1 	TWALAKTLRIELGSQTKD--VTVSLHDVK2S-APSGGTLATALNIWDVFTLKEMKDASSP
AN7293.1  	AYALVKEVREKMSCQTKE--ITGSIYEFK2SSGASGGTLASILTCIEDFSGSQLLESTKP
          	:::*.: :: ::.  *:   :  *:   . .: .****  : :   : *: . : :  .*

MG00127.1 	FALSPVPQPSP--PKDQVSLLTRLTGLRNFPLIGLVTTSLMGGANKPIVERTWGLQQTEP
NCU07563.1	YALSPVPHPNPDLAERRTTWITKVTGLATTPILGLGTTSVAAKTDAAIVQRTWGLLSEAS
FG06758.1 	YAQSPIPHKEP--TRPKSTMLEMILGVRSVSNLGLLTTSIAGTTDVAVVERTWGLLSETP
AN7293.1  	FALAASPPPKD---IPSESFVARILGVRTVRDLGTLTKSPTDFCDMTIIHRSS-------
          	:* :. *  .        : :  : *: .   :*  *.*     : .::.*:        

MG00127.1 	--ALRDYSYGPNFSYNEYNRAHGRLFGFAVHLVINVGLPLLTVLWPLRSLLRLLIPYKSG
NCU07563.1	NKGKEDESYGPRFSFKQYMKPRNWLSGVAIHLGLMVLG--LVMATPFLRKVAKKFVYQPG
FG06758.1 	--SRKEEFYGPNFVWQERMKARNWLHGIFIHWLLIIGGVLLVSVAPLREFLKKRVVQ-PG
AN7293.1  	--TLMPELYGPRFYFRQFLNVRNTFIGILWNYAFLVGTAFLILAP--VRWLVKKAIYAPG
          	        ***.* :.:  . :. : *.  :  : :    *         :       .*

MG00127.1 	EGPSRETSARQKTTFRAIARPD------MPPGSCDKMAFAKADFNG-SMYHM1TGICIAR
NCU07563.1	EGAEEEVAKRDELEYWGVAKPDFGGDEVKEKEEGRKRAFGRCWFRG-PVYYY1TGVLLAE
FG06758.1 	QGASSEDTAKDYVEYRGIAYPD------SEK-QATKAAFGRMWHHG-GMYLL1TAVLLSE
AN7293.1  	SGPTTESGRNDRCEYRAIATPD---------QDTPKRVLGKLKYAGSSMYHF~TGVLMAE
          	.*.  *   .:   : .:* **          .  * .:.:  . *: :*   *.: ::.

MG00127.1 	GAQTILEEYDSLQLKGG--VYTPASLGQGFIDRVGEDGFEIKTKIVKS
NCU07563.1	AAATLLEEESVDEQLGGGGIYTPACLGQPFIDRLDKAGFHFETKIMEE
FG06758.1 	IAATILEDN--VELDGG--SYTPACLGQGLIDRLDKSGLKLDVKTIDV
AN7293.1  	AAMVILENEEKVKRVSGGGIVTPASLGQEYLDRLENNGCRVETQVLEN
          	 * .:**: .  :  .*..  ***.***  :**: : * ....: :.
```
